# Laparoscopic T-tube feeding jejunostomy as an adjunct to staging laparoscopy for upper gastrointestinal malignancies: the technique and review of outcomes

**DOI:** 10.1186/s12893-017-0221-2

**Published:** 2017-03-20

**Authors:** Sze Li Siow, Hans Alexander Mahendran, Chee Ming Wong, Nirumal Kumar Milaksh, Myo Nyunt

**Affiliations:** 1 Department of Surgery, Faculty of Medicine and Health Sciences, Universiti Malaysia Sarawak, 94300 Kota Samarahan, Kuching, Sarawak Malaysia; 2 Department of Surgery, Jalan Hospital, 93586 Kuching, Sarawak Malaysia

**Keywords:** Laparoscopic jejunostomy, Feeding jejunostomy, Tube jejunostomy, Staging laparoscopy, Oesophagogastric cancer

## Abstract

**Background:**

In recent years, staging laparoscopy has gained acceptance as part of the assessment of resectability of upper gastrointestinal (UGI) malignancies. Not infrequently, we encounter tumours that are either locally advanced; requiring neoadjuvant therapy or occult peritoneal disease that requires palliation. In all these cases, the establishment of enteral feeding during staging laparoscopy is important for patients’ nutrition. This review describes our technique of performing laparoscopic feeding jejunostomy and the clinical outcomes.

**Methods:**

The medical records of all patients who underwent laparoscopic feeding jejunostomy following staging laparoscopy for UGI malignancies between January 2010 and July 2015 were retrospectively reviewed. The data included patient demographics, operative technique and clinical outcomes.

**Results:**

Fifteen patients (11 males) had feeding jejunostomy done when staging laparoscopy showed unresectable UGI maligancy. Eight (53.3%) had gastric carcinoma, four (26.7%) had oesophageal carcinoma and three (20%) had cardio-oesophageal junction carcinoma. The mean age was 63.3 ± 7.3 years. Mean operative time was 66.0 ± 7.4 min. Mean postoperative stay was 5.6 ± 2.2 days. Laparoscopic feeding jejunostomy was performed without intra-operative complications. There were no major complications requiring reoperation but four patients had excoriation at the T-tube site and three patients had tube dislodgement which required bedside replacement of the feeding tube. The mean duration of feeding tube was 127.3 ± 99.6 days.

**Conclusions:**

Laparoscopic feeding jejunostomy is an important adjunct to staging laparoscopy that can be performed safely with low morbidity. Meticulous attention to surgical techniques is the cornerstone of success.

## Background

Staging laparoscopy has emerged as an important staging modality for upper gastrointestinal (UGI) malignancies. It is most useful in detecting and confirming nodal involvement and small liver and peritoneal metastases that can potentially alter the prognosis and treatment strategy from curative to palliative intent [1, 2]. The placement of a feeding jejunostomy tube during staging laparoscopy is often necessary to establish enteral feeding when oral intake is not possible or a gastrointestinal obstruction is expected to occur, such as in the presence of unresectable obstructed tumour or advanced metastatic cancer. Patients with severe sarcopenia will also benefit especially if they are to undergo neoadjuvant chemotherapy for down-staging or palliative chemotherapy. The benefits of a feeding jejunostomy to enable improvement of nutrition in those requiring chemotherapy and maintenance of enteral access during the period of profound gastrointestinal toxicity while on chemotherapy cannot be underestimated.

The first laparoscopic technique of feeding jejunostomy was described by O’Regan et al. in 1990 [3]. The technique underwent modifications with several descriptions and commercially available products that facilitated the insertion of feeding tubes were introduced. However, the use of commercially available product increases the cost of the surgery, making it unfavorable in developing countries where health budget is a concern. Thus, we devised a total laparoscopic technique using a T-tube to overcome this limitation. This review describes our initial experience with laparoscopic feeding jejunostomy with its technical details when used as an adjunct to staging laparoscopy.

## Methods

A retrospective review of all patients (15 patients) who underwent laparoscopic feeding jejunostomy during staging laparoscopy for UGI malignancy between March 2010 and July 2015 was performed. The indications for feeding jejunostomy were: 1) Metastatic disease with peritoneal nodules or 2) Locally advanced carcinoma requiring neoadjuvant therapy for down-staging. The decision for feeding jejunostomy or palliative gastrojejunostomy bypass procedure in patients with metastatic cancer was based on the degree of tumor infiltration of the stomach wall. Palliative gastrojejunostomy will be the preferred option in patients with gastric outlet obstruction. However, patients with linitis plastica or gastric inlet obstruction; feeding jejunostomy was performed. The data analyzed included demographics, American Society of Anesthesiologists (ASA) score, body mass index (BMI), types of malignancy, indications for feeding jejunostomy, operative technique, operative time, length of hospitalization and operative outcomes. The study was approved by the hospital ethics committee and Director-General of Health of Malaysia. Preoperative computed tomography (CT) scan of the abdomen and pelvis was the routine method of pre-operative staging. Prophylactic antibiotic was given intravenously during induction of anesthesia. Clear fluid was started via the feeding tube postoperatively on the day of surgery. Enteral milk feeding was started on the first postoperative day, employing a standard protocol outlined in the department. The feed was administered as a continuous infusion commencing at 30 ml/h for 3 h with an hour’s break between feeds. The feeds were gradually increased to 100–150 ml/h as tolerated. Patients were allowed oral free fluids as tolerated and were discharged with out-patient appointments. Patients must have established full enteral feeds and no major tube-related complications. The T-tube remained in-situ until the end of patients’ lifespan or removed when patients were able to tolerate sufficient diet containing solid food or at their request.

Following discharge from the ward, patients were reviewed once every 3 months for the first 2 years, then every 6 months for the following 3 years. There was no loss of patients to follow-up during the study period. Patients or the next of kin were contacted in the event of a missed clinic appointment.

Complications were broadly classified into early (those occurring within 30 days of jejunostomy placement) and late (those occurring ≥30 after the procedure). These complications were either tube-placement related, or feed related (bloating, diarrhoea and abdominal colic). Complications were further categorized as minor (catheter occlusion, catheter dislodgement, pericatheter leakage, tube site infection, and feed intolerance) or major (bleeding requiring blood transfusion, intestinal obstruction, peritonitis, volvulus, aspiration and any potentially life threatening adverse event requiring the need of a surgical or radiologic intervention).

### Operative technique

**Fig. 1:**
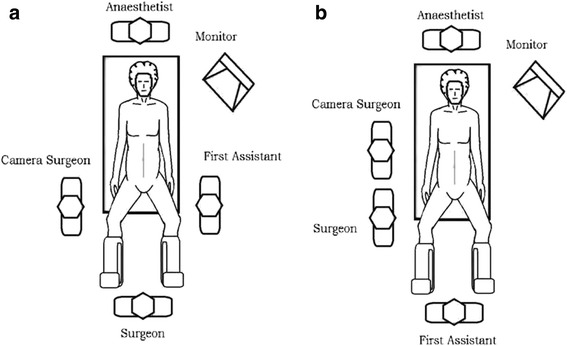
**a** Team position for staging laparoscopy. **b** Team position for laparoscopic feeding jejunostomy

**Fig. 2:**
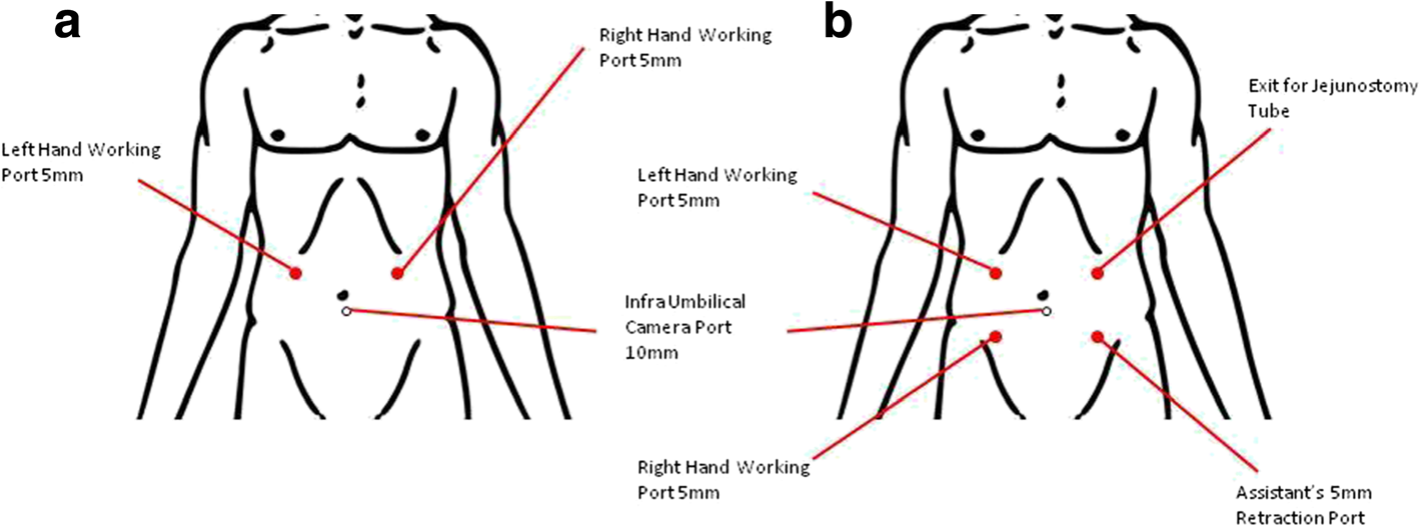
**a** Port placement for staging laparoscopy. **b** Port placement for laparoscopic feeding jejunostomy

The patient is positioned in modified lithotomy with both legs supported in padded yellow fin (Allen Medical, USA) stirrups. Figure 1a illustrates the position of the surgeon, camera surgeon and assistant during staging laparoscopy and Fig. 1b demonstrates the team position during laparoscopic feeding jejunostomy. Abdominal access is performed using Hasson’s technique with pneumoperitoneum established via a 10-mm infraumbilical port. Two 5-mm ports are placed in the right and left mid-clavicular line to facilitate manipulation of the bowels and stomach (Fig. 2a). We routinely perform staging laparoscopy in a reverse TNM manner, evaluating the presence of distant metastasis first, followed by extent of nodal infiltration and finally the extent of tumor infiltration itself. Any suspected peritoneal nodules or lymph nodes are biopsied. Ascites, if present, would be aspirated and sent for cytological evaluation.

**Fig. 3:**
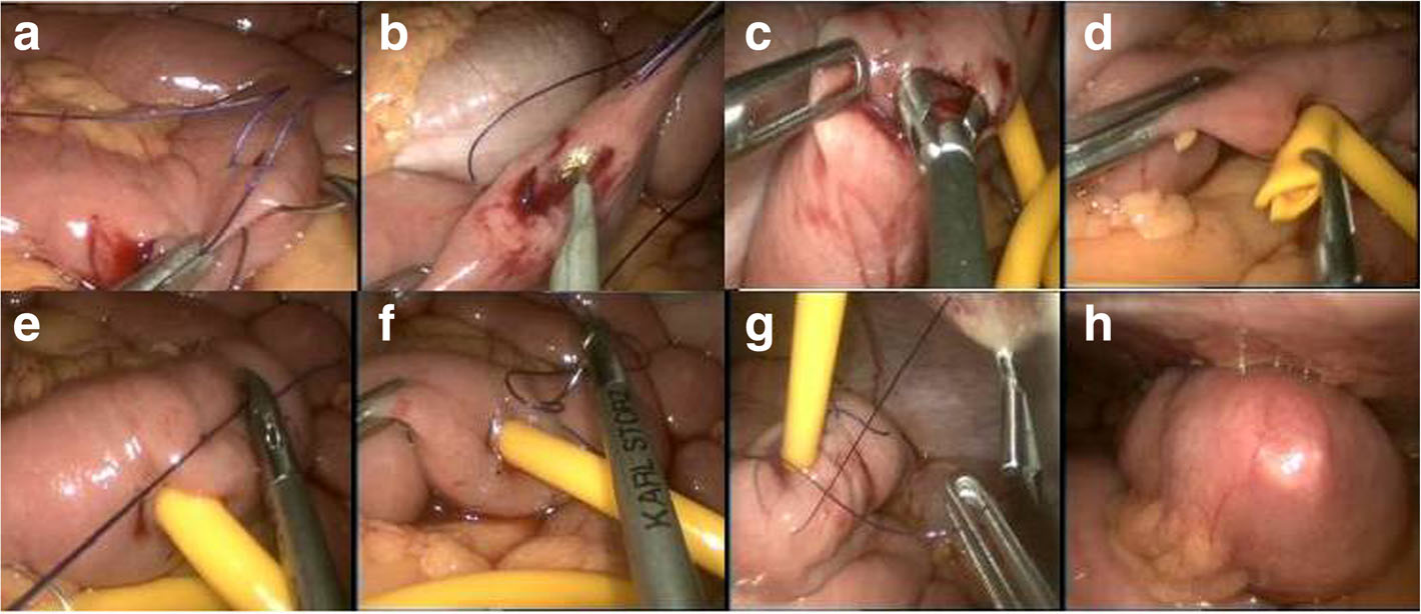
Jejunostomy technique **a** First layer of purse-string suture of jejunostomy tube using polyglactin 910 3/0 suture. **b** Enterotomy done with hook. **c** Enterotomy widened using Maryland dissector. **d** Insertion of T-tube into enterotomy. **e** First layer of purse-string suture knot secured. **f** Second layer of purse-string made using the remaining polyglactin 910 3/0 suture. **g** Transfascial suturing with suture passer (thread grasper) introduced through the same 2-mm stab incision in a different track. **h** T-tube flushed with normal saline to check for patency and leak

**Fig. 4:**
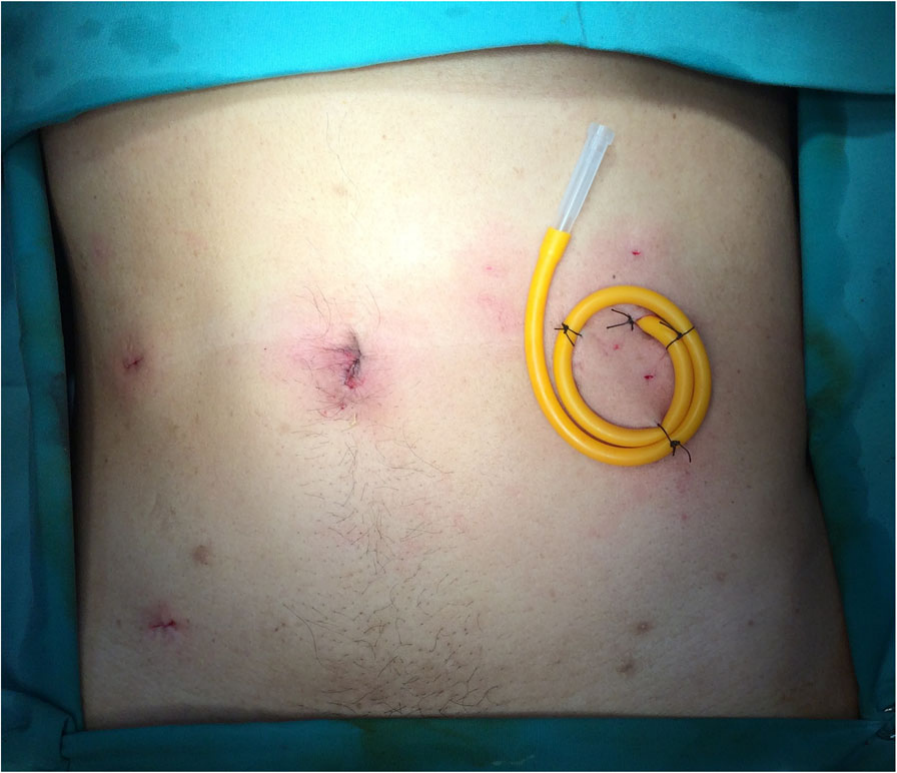
Final appearance of the T-tube jejunostomy against the patient’s abdominal wall

After staging laparoscopy has determined the tumour is unresectable, the surgeon repositions himself to the right side of the patient next to the camera surgeon, and the assistant then stands between the patient’s legs. Figure 2b illustrates the port position for laparoscopic feeding jejunostomy. The ligament of Treitz is first identified, and then a loop of proximal jejunum approximately 30 to 40 cm distal to the ligament is selected. A first layer of purse-string suture using polyglactin 910 is placed on the antimesenteric border using a laparoscopic needle holder (Fig. 3a). An enterotomy is made with hook and widened using a Maryland dissector (Fig. 3b, c). Then, a T-tube (Teleflex Medical, Kernen, Germany), size 6 mm, with its back wall hemisected is introduced into the abdomen through the 10-mm port, and inserted into the enterotomy using Maryland (Fig. 3d). Once the arms of the T-tube (Teleflex Medical, Kernen, Germany) are successfully placed into the jejununal lumen, the purse-string suture is tightened and knotted (Fig. 3e). A second ring of purse-string suture is done utilizing the remaining length of the initial suture and subsequently knotted (Fig. 3f). Transabdominal fixation of the jejunum is performed using a 2-0 polypropylene suture placed approximately 2 cm proximal and distal to the T-tube (Teleflex Medical, Kernen, Germany), taking a seromuscular bite into the jejunal wall. Subsequently, the needle is removed with both ends of suture brought out onto the surface of abdomen using a suture passer introduced through the same 2-mm stab incision in a different track (Fig. 3g). Once the tube is brought out through 5-mm port site, traction is applied to the two free ends of transabdominal fixation sutures to approximate the jejunum onto the peritoneal surface of the abdominal wall. The sutures are tied with the knot secured anterior to the fascia and buried in the subcutaneous tissue. Finally, the tube is flushed with normal saline solution to check the flow and ensure no leak (Fig. 3h). Figure 4 illustrates the final appearance of T-tube against the abdominal wall.

The patients were reviewed by the nutritional support team postoperatively. Tube feeding commenced from postoperative day one via an infusion pump at a rate of 30 ml/h. Feeding was gradually increased over the next 2–3 days. A feeding pump was used during the initial phase of enteral nutrition until bolus feeds were tolerated.

## Results

Fifteen patients were enrolled in this study and the results are summarized in Table 1. The mean age of the patients was 63.3 ± 7.3 years, and 11 patients (73.3%) were male. Eight patients (53.3%) had gastric carcinoma, four (26.7%) had oesophageal carcinoma and three (20%) had cardio-oesophageal junction carcinoma. The indications for feeding jejunostomy during staging laparoscopy were as follows: Palliative setting in non-resectable or metastatic carcinoma with obstructive symptoms (60.0%) and locally advanced carcinoma for neo-adjuvant chemotherapy (40.0%). Laparoscopic feeding jejunostomy was performed successfully for all patients. There were no intra-operative complications occurred as a consequence of the tube insertion technique. The mean operative time was 66.0 ± 7.4 min. Enteral feeding was commenced the next day for all patients after reviewed by nutrition support team and the appropriate polymeric formula decided. The aim was to achieve and establish adequate enteral feeding (more than 50% total energy requirement) within 3 days. Three patients did not achieve this target due to abdominal distension and was self-limiting. The mean postoperative stay was 5.6 ± 2.2 days. There were seven cases of minor late complications, including four cases of minor leak and excoriation around the T-tube and three cases of tube dislodgement. However, all were managed expectantly without the need for reoperation. The mean duration of feeding tube was 127.3 ± 99.6 days.

**Table 1:** Demographics and surgical outcomes of patients who underwent laparoscopic T-tube feeding jejunostomy

Variable	Value	Range
Number of patients	15	
Age (years, mean ± SD)	63.3 ± 7.3	48.0–73.0
Gender (male:female)	11:4	
Body mass index	19.8 ± 2.8	15.0–23.5
ASA		
1	2	
2	12	
3	1	
Operative time (minutes, mean ± SD)	66.0 ± 7.4	55.0–80.0
Postoperative hospital stay (days, mean ± SD)	5.6 ± 2.2	2.0–9.0
Operative-related complications	0	
Conversion to laparotomy	0	
Early complications		
Minor^a^	3	
Major^b^	0	
Late complications		
Minor^c^	7	
Major^b^	0	

^a^Minor early complications: 3 patients with feed intolerance

^b^Major complications: tube-related complications requiring re-operation

^c^Minor late complications: 4 minor leaks and skin excoriation & 3 tube dislodgement

## Discussion

This study shows that laparoscopic feeding jejunostomy can be performed safely with no significant morbidity as an adjunct to staging laparoscopy. The procedure requires only standard basic laparoscopic instruments such as laparoscopic hook, Maryland forceps and monopolar electrocautery. The use of T-tube is an inexpensive alternative to commercially available feeding tubes.

Since the first description of the use of jejunostomy tube in 1891 by Witzel [4], a vast majority of patients with UGI malignancies requiring nutritional support have successfully undergone open jejunostomies. However, the open technique is associated with increased operative morbidity and hospital stay [5]. A laparoscopic approach is ideal as it not only confers the advantages of minimally invasive techniques but avoids inflicting an additional surgical scar as one of the port sites can be used as the exit wound for the feeding tube with lower rates of surgical site infection.

There are considerable variations in techniques described in the literature for performing laparoscopic feeding jejunostomy. In general, either the total laparoscopic or laparoscopic-assisted techniques have been employed [6]. The most common technique for total laparoscopic placement of feeding catheter is the Seldinger technique [1, 7, 8]. It is a technique commonly involves a commercial product [9–11], with percutaneous placement of feeding catheter performed after the bowel is secured to the abdominal wall, using a combination of needle, wire, dilator, stent and feeding tubes [1, 7, 8]. Different combination techniques have been described [12, 13]. Laparoscopic-assisted techniques involve exteriorizing the jejunum through a small abdominal incision or one of the trocar openings [14] to allow extracorporeal enterotomy and placement of the catheter. Total laparoscopic technique is superior as it avoids minilaparotomy incision but has the disadvantage of requiring intracorporeal suturing [6]. Our technique is a total laparoscopic technique that does not requires percutaneous jejunostomy kit. It mimicks the open technique with the initial placement of the feeding tube, followed by the withdrawal of the tube through a left abdominal port site and finally fixation of the jejunum to the abdominal wall via transabdominal sutures.

There are three techniques described to secure the entry of the feeding jejunostomy tube into the jejunum: a purse-string suture, the Stamm inverting style or a Witzel tunnel [13]. We adopt the purse-string suture method as we feel that it is an easier option laparoscopically as compared to the Witzel and Stamm techniques. The Stamm technique, initially described for gastric access and later adopted for enteral access, incorporates both the purse-string suture around the enterotomy site and inverting stitch of jejunal wall around the tube to the overlying peritoneum. It requires some degree of finesse in order to place an inverting stitch laparoscopically through the abdominal wall, a maneuver that requires a pronounced supination-pronation of the wrist to drive the needle through the abdominal wall. The Witzel technique involves creating a short serosal tunnel with imbricating sutures over the tube and along the long axis of the bowel. One study that favour Witzel tunnel indicated that such technique reduces the incidence of severe surgical site infections and the rate of late jejuno-cutaneous fistulation [13]. Performing Stamm and Witzel techniques laparoscopically, though feasible, can be technically challenging and time consuming when done exactly like in the open technique [7]. Taking too much of the jejunum while constructing Witzel tunnel could also lead to luminal obstruction at the catheter insertion site [15]. Dislodgement of the catheter from a Witzel tunnel collapses the tunnel and does not allow easy placement of catheter. On the contrary, a straight passage from the enterotomy to the anterior abdominal wall is important as it gives a straight trajectory that allows easy replacement of a catheter in the event of dislodgement. The rationale of the purse-string sutures is to create a seal around the jejunal catheter. A second purse-string may be over-elaborative or unnecessary. However, we maintain the practice as we did not encounter any case of intraperitoneal leakage of jejunal content. In addition, it is a much simpler procedure than the Stamm technique of additional inverting stitch. Transfascial suturing aligns the jejunum to the parietal peritoneum and minimizes the risk of volvulus.

Different techniques of anchoring the jejunum to the anterior abdominal wall have been described, either in the forms of transfascial sutures (transabdominal sutures [8, 10] or T-fasteners [5, 16]) or intracorporeal sutures [13, 17]. The transabdominal sutures (3–4 in number) are usually placed in a diamond configuration, incorporating the seromuscular layer of the jejunal wall and the anterior abdominal wall [6]. The free ends of the suture are brought out onto the surface of abdomen using a thread gasper and the two threads are tied with the knot secured at the fascial layer [15]. Alternatively, the suture could be tied over bolster placed on the skin to prevent skin damage from the suture [8, 11]. The T-fastener, originally developed for fixation of stomach to the anterior abdomen in laparoscopic gastrostomy, consists of a nylon suture attached to a metal T-bar, is introduced percutaneously and dislodged into the jejunal lumen from the slotted needle by the stylet [5, 16, 18]. Its placement over antimesenteric jejunal wall usually follows a diamond configuration [5, 16]. Our technique differs to the conventional 3–4 sutures diamond or triangular configuration. We believe that 2 sutures suffice in aligning the jejunum against the abdominal wall after the purse-string sutures have secured the tube snugly into the enterotomy.

In terms of outcomes, our initial results demonstrate that the technique of laparoscopic T tube feeding jejunostomy can be performed as an adjunct to staging laparoscopy without any increase in peri-operative morbidity. The main technical challenge encountered during this procedure was the insertion of the T tube into the enterotomy. Prior to insertion, it is important to remove the back wall of the horizontal limbs of the T-tube in order to prevent clogging as well as allowing guide-wire access for tube exchange^i^. Additionally, we cut the horizontal limbs into two unequal ends. Our insertion technique entails initial widening of the enterotomy using Maryland forceps and inserting the long end first followed by the short end. We feel that the technical dexterity required for tube insertion will be improved once the procedure is performed on a regular basis.

The complication rates reported for feeding jejunostomy in the literature is variable, with an overall rate between 1.5 and 37% [19]. We compared our data and complication with 12 selected series in the literature that report on the outcomes of laparoscopic feeding jejunostomy (Table 2). The rate for conversion to open surgery ranges from 0 to 12.5% [11, 13], minor complications ranges from 5.3 to 32.1% [11, 20], major complications ranges from 0 to 10.7% [10, 11, 21] and mortality ranges from 0 to 11.1% [5]. The reported complications include wound infection [6, 22]; catheter dislodgement [6, 13, 23]; occlusion [13, 23]; pericatheter leak with generalized peritonitis [24]; aspiration pneumonia [22, 24]; small bowel necrosis [23, 25]; small bowel obstruction [13, 22, 23]; pneumatosis intestinalis [23, 26]; abdominal wall infection [13, 23]; fistula [13, 23]; volvulus [23]; and death [23, 25]. Myers JG et al. [23] presented an analysis of complications in a large series of patients involving insertion of needle catheter jejunostomy at the time of laparotomy as an adjunct for a variety of reasons. In their series of 2022 patients, the authors found a low complication rate of 1.5% and concluded that learning curve and case volume, in addition to meticulous attention to operative details, are the important factors accounting for it. Their observation was substantiated by their review of series with more than 150 catheter placements in which the reported complication rate did not exceed 3% [23]. On the contrary, two retrospective cohort studies on laparoscopic feeding jejunostomy with case volume in excess of 150 reported an overall complication rate of 9.8–12.7% [13, 21]. In addition, most authors do not have series larger than 50 patients, and such low complication rates could not be replicated. Nevertheless, under-reporting of particularly minor complications as compared to major complications and mortality can occur in retrospective analysis and some authors argued that the safety of jejunostomy tube placement should be assessed primarily in terms of major complications requiring surgical intervention or resulting in death [21]. Han-Geurts IJ et al. in their systematic review of laparoscopic feeding jejunostomy involving a series of 384 patients detected a complication rate of 17% which was comparable to that of open surgery [6]. From their analysis, wound infection and tube dislodgement were the most common complications [6]. Similar findings were observed in the current series that reported a late complication rate of 46.7% (seven patients). However, no patients had serious complications that required surgical intervention and there was no death associated with the procedure. The mean hospital stay after the surgery was 5.5 days. The delay in discharge was mainly because of the institution of the enteral feeding, awaiting referral to oncologists and logistic issues. Four patients (26.7%) developed late complication of skin excoriation around the tubing of which three were managed conservatively with dressing and antibiotics, and one had a change of catheter. Three patients (20%) had catheter dislodgement which was successfully replaced with Foley catheter at bedside.

**Table 2:** Comparison of selected studies on laparoscopic feeding jejunostomy in cohorts of 10 or more patients

Author	No.of Cases	Indication for placement	Operative Techniques (total laparoscopic/laparoscopic aided)	Tube-related complications (Minor/Major)	Feed-related gastrointestinal symptoms	Conclusions
Sangster W et al. [9]	23	Various indications	Total laparoscopic using a 10-French jejunostomy catheter kit	Minor complications (*n* = 2, 8.7%): superficial skin breakdown around the tube (*n* = 2). Major complications (*n* = 1, 4.3%): superficial abscess around the tube requiring I & D. One unrelated death.	NM	No procedure related complications. A valuable addition to the surgeon’s options for obtaining enteral access.
Grondona P et al. [10]	18	Part of staging laparoscopy for esophagogastric cancer	Total laparoscopic using a dedicated feeding jejunostomy kit	Minor complications (*n* = 3, 16.7%): tube dislodged (*n* = 1), leakage with wound infection (*n* = 1) & wound infection (*n* = 1). No major complications.	NM	A safe and reliable technique. A useful adjunct to staging laparoscopy for esophagogastric cancer.
Allen JW et al. [15]	35	Various indications	Total laparoscopic using a 16 French T-tube	Minor complications (*n* = 4, 11.4%): wound infection (*n* = 2) & leakage (*n* = 2) Major complications (*n* = 1, 2.9%): intractable pain requiring laparotomy	NM	Safe technique with no significant morbidity or mortality
Ben-David K et al. [21]	153	Prior to definitive minimally invasive esophagectomy	Total laparoscopic using a 16-French T-tube	Minor complications (*n* = 15, 9.8%): superficial wound infection (*n* = 4), dislodgement (*n* = 2), leak (*n* = 4) & clogging (*n* = 5). No major complications.	NM	A feasible and safe technique in one of the largest series of laparoscopic feeding jejunostomy tube for esophageal cancer patients.
Mistry RC et al. [20]	19	Oesophageal resection	Total laparoscopic using a 12-French T-tube	Minor complications (*n* = 1, 5.3%): extraperitoneal leakage of feeds due to a damaged vertical limb of the T-tube.	NM	An easy, inexpensive technique that does not require specialized equipment or feeding tubes.
Senkal M et al. [12]	80	Primary or recurrent tumors of the upper gastrointestinal tract	Total laparoscopic using a 9-French jejunostomy catheter kit	Minor complications (*n* = 7, 8.8%): leakage (*n* = 2), tube occlusion (*n* = 3) & dislodgement (*n* = 2). Major complications (*n* = 1, 1.3%): abscess at the insertion site requiring drainage.	NM	A safe and effective technique. Does not require special equipment such as T-fasteners, or transabdominal suturing.
Heath EI et al. [1]	59	Part of the staging laparoscopy for esophageal cancer	Total laparoscopic using a 10-French jejunostomy tube	Only major complications reported (*n* = 2, 3.4%): perforation of the small bowel requiring laparotomy and small bowel resection (*n* = 1) & intraoperative pulmonary oedema secondary to aortic valve stenosis (*n* = 1).	NM	Reported only two major complications with only one related to the procedure of laparoscopic feeding jejunostomy. Minor complications were not reported.
Hotokezaka M et al. [11]	32	Various indications	Total laparoscopic using an 18-French Silastic duallumen feeding tube	Conversion to open (*n* = 4, 12.5%). Minor complications (*n* = 9, 32.1%): dislodgement (*n* = 3), obstruction (*n* = 2) & leakage/wound erythema (*n* = 4). Major complications (*n* = 3, 10.7%): dislodgement (*n* = 1) & aspiration pneumonia (*n* = 2). Death within 30 days (*n* = 3, 10.7%): aspiration pneumonia and respiratory distress (*n* = 1) & unrelated death (*n* = 2).	Four patients (14.2%) had nausea and one (3.6%) abdominal cramp.	Safe procedure. High morbidity is usually related to preexisting disease. Previous abdominal surgery is not necessarily a contraindication.
Jenkinson AD et al. [17]	43	Part of the laparoscopic staging for esophagogastric cancer	Total laparoscopic using a 6-French infant feeding catheter (Vygon)	Minor complications (*n* = 11, 25.6%): dislodgement (*n* = 5), blockage (*n* = 4) & connector breakage (*n* = 2). Major complications (*n* = 1, 2.3%): Dislodgement requiring laparoscopic replacement.	NM	A safe and simple technique that adds little to the morbidity and cost of managing patients with esophagogastric cancers.
Pili D, et al. [30]	25	Patients undergoing major surgery for esophageal cancer	Total laparoscopic using 8- French jejunostomy catheter kit.	Minor complications (*n* = 3, 12.0%): chronic catheter occlusion (*n* = 2) & slippage (*n* = 1). No major complications.	NM	No procedure related morbidity or mortality. A feasible procedure with the use of autoadjustable sutures to overcome the limitation of the laparoscopic handling.
Duh QY et al. [5]	36	Various indications (a multicentre study)	Total laparoscopic using jejunostomy catheter kit and T-fasteners.	Conversion to open (*n* = 3, 8%). Minor complications (*n* = 6, 16.7%): wound erythema or infection (*n* = 3) & dislodgement (*n* = 3). Major complications (*n* = 3, 8.3%): volvulus (*n* = 1) & dislodgement (*n* = 2). Death (*n* = 4, 11.1%): unrelated to procedure.	NM	A safe and effective technique when done by experienced laparoscopic surgeons. Serious complications are rare.
Young MT et al. [13]	299	Various indications with majority for esophagogastric cancer	Total laparoscopic using 10-French jejunostomy catheter kit	No conversion to open surgery. ^a^Early complications (*n* = 12, 4.0%): dislodgement (*n* = 3), clogging (*n* = 3), intraperitoneal displacement (*n* = 2), broken tube (*n* = 1), rectus sheath hematoma (*n* = 1) & abdominal wall site infection (*n* = 2). Late complications (*n* = 26, 8.7%): small bowel obstruction (*n* = 1), jejunal fistula (*n* = 11), dislodgement (*n* = 10) & broken or cogged tube (*n* = 4). Mortality (*n* = 1, 0.3%): unrelated to procedure.	NM	A safe and feasible technique. Associated with a low rate of small bowel obstruction and no intraabdominal catheter-related infection.
Present series	15	Part of the staging laparoscopy for upper gastrointestinal malignancies	Total laparoscopic using 18-French T-tube	Minor complications (*n* = 7, 46.7%): Skin excoriation around tubing (*n* = 4) & catheter dislodgement (*n* = 3). No major complications.	Three patients (20.0%) had feed intolerance.	A safe, cost-effective technique with no procedure related complications.

*I & D* incision & drainage, *NM* not mentioned. ^a^The authors divided the complications into early (30-day) and late (˃30 day), and did not fully specify the treatment action for each individual complications and hence not able to differentiate between minor and major complications

Feeding jejunostomy is a simple procedure yet an important adjunct to staging laparoscopy. With the aim of achieving early enteral feeding and a reduction in postoperative morbidity, any complications arising from the procedure will jeopardize its benefits as it will incur additional costs and delay subsequent oncologic treatment. Meticulous attention to tube placement technique remains a sine qua non to limit complication rates. Dislodgement of the tube can be avoided by attention paid to the technique of securing and confirming catheter placement prior to usage [23]. Appropriate fixation of jejunum to the parietal peritoneum avoids migration of tube to the abdominal cavity [27], and the occurrence of small bowel volvulus or obstruction at the jejunostomy site [5, 23]. Our technique of double purse-string suturing ensures that the tube fits snugly in the small bowel, eliminating the risk of leakage of jejunal content. In addition, the T configuration of the tube prevents the risk of accidental tube dislodgement unless the tube is forcefully jerked. Transfascial suturing aligns the jejunum to the parietal peritoneum, preventing the bowel from falling away from the anterior abdominal wall. Our technique may appear to be more demanding than those described in the literature but it can be mastered from repeated practice. The time invested in perfecting the technique is rewarded with a favorable outcome as reflected in our series showing no leak or dislodgement. Prior to initiating enteral feeding, some authors perform a contrast study a day after the procedure prior to confirm the patency and intraluminal position of the tube [12, 23]. However, we typically flush the feeding catheter with normal saline to check its position and for any leak under laparoscopic visualization intra-operatively.

A T-tube has several advantages over other types of tubes. Firstly, the T configuration of the tubing is resistant to accidental dislodgement of the tube, reducing the risk of peritonitis. Secondly, the soft latex T-tube has less risk of intestinal perforation as compared to stiffer jejunostomy tubes and encourages the early formation of a fistulous tract [28]. This enables safe and easy replacement in the event of dislodgement [28]. In addition, a T-tube obviates the risk of bowel obstruction as it is generally smaller than other types of tube and it does not require an insufflated balloon to maintain its position in the bowel lumen. The insertion of the tube under direct vision and confirmation of position and non-leakage at the end of procedure eliminates the need for radiological confirmation. Balloon devices have been known to cause bowel obstruction due to overfilling of the balloon which can also cause pressure necrosis of the bowel wall.

Feeding intolerance is demonstrated when the patient developed feeding-related abdominal symptoms such as abdominal distension and diarrhea [19]. The degree of enteral tolerance varies in different studies and the frequency ranges from 5 to 35% [29]. However, it is often self-limited and can be corrected by adjusting the infusion rate and concentration of the feed or temporary cessation of feeding [29]. Three patients in our series had feed intolerance and did not achieve the target calorie and protein requirements within three days but the abdominal distension was self-limiting and resolved.

**Fig. 5:**
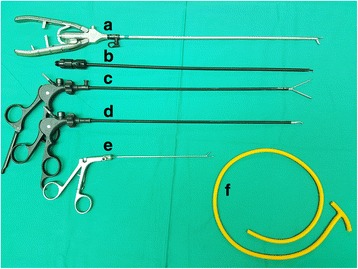
Surgical instruments and the T-tube device needed to perform the procedure **a** Laparoscopic needle holder. **b** Laparoscopic L-hook. **c** Laparoscopic Johan grasper. **d** Laparoscopic Maryland dissecting forceps. **e** Laparoscopic suture passer. **f** T-tube

The main limitations of this study are its retrospective nature of review and small number of cases. However, our innovative technique of totally laparoscopic placement of jejunal T-tube is cost-effective since it requires only standard basic laparoscopic instruments (Fig. 5) and can be performed safely without procedure related morbidity or mortality. In comparison to three other similar studies [15, 20, 21], our technique was unique in terms of the use of a larger-sized T-tube, the double purse-string technique and the transfascial suturing with the suture passer for bowel alignment.

## Conclusions

In conclusion, our experience with laparoscopic feeding jejunostomy as an adjunct to staging laparoscopy demonstrates that it is a safe and feasible technique. Our inexpensive modification using a T-tube is safe with no immediate post-operative complications or mortality resulting from the procedure. It enables nutritional supplementation for patients with metastatic UGI malignancies as well as patients who require neoadjuvant therapy to downstage their tumours. The overall incidence of complications in our series may seem unacceptably high but the complications were all minor and were managed expectantly.

## Abbreviations

ASA: American society of anesthesiologists
BMI: Body mass index
CT: Computed tomography
TNM: Tumour nodes metastasis.
UGI: Upper gastrointestinal

## References

[1] Heath EI, Kaufman HS, Talamini MA, Wu TT, Wheeler J, Heitmiller RF (2000). The role of laparoscopy in preoperative staging of esophageal cancer. Surg Endosc.

[2] D’Ugo DM, Persiani R, Caracciolo F, Ronconi P, Coco C, Picciocchi A (1997). Selection of locally advanced gastric carcinoma by preoperative staging laparoscopy. Surg Endosc.

[3] O’Regan PJ, Scarrow GD (1990). Laparoscopic jejunostomy. Endoscopy.

[4] Witzel O (1891). Zur teknik der magenfistelanlegung. Centralbl Chir.

[5] Duh QY, Senokozlieff-Englehart AL, Siperstein AE, Pearl J, Grant JP, Twomey PL (1995). Prospective evaluation of the safety and efficacy of laparoscopic jejunostomy. West J Med.

[6] Han-Geurts IJ, Lim A, Stijnen T, Bonjer HJ (2005). Laparoscopic feeding jejunostomy: a systematic review. Surg Endosc.

[7] Allen JW, Spain DA (2001). Open and laparoscopic surgical techniques for obtaining enteral access. Tech Gastrointest Endosc.

[8] Schirmer BD. Laparoscopic placement of jejunostomy tube. In: Soper NJ and Scott-Connor CEH, editors. The SAGES Manual: Volume 1 Basic Laparoscopy and Endoscopy, Springer Science + Business Media. New York: Springer-Verlag. 2012. p. 379–87.

[9] Sangster W, Swanstrom L (1993). Laparoscopic-guided feeding jejunostomy. Surg Endosc.

[10] Grondona P, Andreani SM, Barr N, Singh KK (2005). Laparoscopic feeding jejunostomy technique as part of staging laparoscopy. Surg Laparosc Endosc Percutan Tech.

[11] Hotokezaka M, Adams RB, Miller AD, McCallum RW, Schirmer BD (1996). Laparoscopic percutaneous jejunostomy for long term enteral access. Surg Endosc.

[12] Senkal M, Koch J, Hummel T, Zumtobel V (2004). Laparoscopic needle catheter jejunostomy: modification of the technique and outcome results. Surg Endosc.

[13] Young MT, Troung H, Gebhart A, Shih A, Nguyen NT (2016). Outcomes of laparoscopic feeding jejunostomy tube placement in 299 patients. Surg Endosc.

[14] Gedaly R, Briceno P, Ravelo R, Weisinger K (1997). Laparoscopic jejunostomy with an 18-mm trocar. Surg Laparosc Endosc.

[15] Allen JW, Ali A, Wo J, Bumpous JM, Cacchione RN (2002). Totally laparoscopic feeding jejunostomy. Surg Endosc.

[16] Duh QY, Way LW (1993). Laparoscopic jejunostomy using T-fasteners as retractors and anchors. Arch Surg.

[17] Jenkinson AD, Lim J, Agrawal N, Menzies D (2007). Laparoscopic feeding jejunostomy in esophagogastric cancer. Surg Endosc.

[18] Murayama KM, Johnson TJ, Thompson JS (1996). Laparoscopic gastrostomy and jejunostomy are safe and effective for obtaining enteral access. Am J Surg.

[19] Han-Geurts IJ, Hop WC, Verhoef C, Tran KT, Tilanus HW (2007). Randomized clinical trial comparing feeding jejunostomy with nasoduodenal tube placement in patients undergoing oesophagectomy. Br J Surg.

[20] Mistry RC, Mehta SS, Karimundackal G, Pramesh CS (2009). Novel cost-effective method of laparoscopic feeding-jejunostomy. J Minim Access Surg.

[21] Ben-David K, Kim T, Caban AM, Rossidis G, Rodriguez SS, Hochwald SN (2013). Pre-therapy laparoscopic feeding jejunostomy is safe and effective in patients undergoing minimally invasive esophagectomy for cancer. J Gastrointest Surg.

[22] Weltz CR, Morris JB, Mullen JL (1992). Surgical jejunostomy in aspiration risk patients. Ann Surg.

[23] Myers JG, Page CP, Stewart RM, Schwesinger WH, Sirinek KR, Aust JB (1995). Complications of needle catheter jejunostomy in 2,022 consecutive applications. Am J Surg.

[24] Cogen R, Weinryb J, Pomerantz C, Fenstemacher P (1991). Complications of jejunostomy tube feeding in nursing facility patients. Am J Gastroenterol.

[25] Smith-Choban P, Max MH (1988). Feeding jejunostomy: a small bowel stress test?. Am J Surg.

[26] Smith CD, Sarr MG (1991). Clinically significant pneumatosis intestinalis with postoperative enteral feedings by needle catheter jejunostomy: an unusual complication. JPEN J Parenter Enteral Nutr.

[27] Tapia J, Murguia R, Garcia G, de los Monteros PE, Onate E (1999). Jejunostomy: techniques, indications, and complications. World J Surg.

[28] Thodiyil PA, El-Masry NS, Peake H, Williamson RC (2004). T-tube jejunostomy feeding after pancreatic surgery: a safe adjunct. Asian J Surg.

[29] Wani ML, Ahangar AG, Lone GN, Singh S, Dar AM, Bhat MA (2010). Feeding jejunostomy: does the benefit overweight the risk (a retrospective study from a single centre). Int J Surg.

[30] Pili D, Ciotola F, Riganti JM, Badaloni A, Nieponice A (2015). Autoadjustable sutures and modified seldinger technique applied to laparoscopic jejunostomy. World J Surg.

